# Value of radiomics and deep learning feature fusion models based on dce-mri in distinguishing sinonasal squamous cell carcinoma from lymphoma

**DOI:** 10.3389/fonc.2024.1489973

**Published:** 2024-11-21

**Authors:** Ziwei Zhang, Duo Zhang, Yunze Yang, Yang Liu, Jianjun Zhang

**Affiliations:** ^1^ Department of Radiology, Baoding First Central Hospital, Baoding, China; ^2^ Department of Postgraduate, Chengde Medical University, Chengde, China

**Keywords:** sinonasal, squamous cell carcinoma, lymphoma, radiomics, deep learning

## Abstract

**Problem:**

Sinonasal squamous cell carcinoma (SNSCC) and sinonasal lymphoma (SNL) lack distinct clinical manifestations and traditional imaging characteristics, complicating the accurate differentiation between these tumors and the selection of appropriate treatment strategies. Consequently, there is an urgent need for a method that can precisely distinguish between these tumors preoperatively to formulate suitable treatment plans for patients.

**Methods:**

This study aims to construct and validate ML and DL feature models based on Dynamic Contrast-Enhanced (DCE) imaging and to evaluate the clinical value of a radiomics and deep learning (DL) feature fusion model in differentiating between SNSCC and SNL. This study performed a retrospective analysis on the preoperative axial DCE-T1WI MRI images of 90 patients diagnosed with sinonasal tumors, comprising 50 cases of SNSCC and 40 cases of SNL. Data were randomly divided into a training set and a validation set at a 7:3 ratio, and radiomic features were extracted. Concurrently, deep learning features were derived using the optimally pre-trained DL model and integrated with manually extracted radiomic features. Feature sets were selected through independent samples t-test, Mann-Whitney U-test, Pearson correlation coefficient and LASSO regression. Three conventional machine learning (CML) models and three DL models were established, and all radiomic and DL features were merged to create three pre-fusion machine learning models (DLR). Additionally, a post-fusion model (DLRN) was constructed by combining radiomic scores and DL scores. Quantitative metrics such as area under the curve (AUC), sensitivity, and accuracy were employed to identify the optimal feature set and classifier. Furthermore, a deep learning-radiomics nomogram (DLRN) was developed as a clinical decision-support tool.

**Results:**

The feature fusion model of radiomics and DL has higher accuracy in distinguishing SNSCC from SNL than CML or DL alone. The ExtraTrees model based on DLR fusion features of DCE-T1WI had an AUC value of 0.995 in the training set and 0.939 in the validation set.The DLRN model based on the fusion of predictive scores had an AUC value of 0.995 in the training set and 0.911 in the validation set.The DLRN model based on the fusion of predictive scores had an AUC value of 0.995 in the training set and 0.911 in the validation set.

**Conclusion:**

This study, by constructing a feature integration model combining radiomics and deep learning (DL), has demonstrated strong predictive capabilities in the preoperative non-invasive diagnosis of SNSCC and SNL, offering valuable information for tailoring personalized treatment plans for patients.

## 1 Introduction

Sinonasal squamous cell carcinoma (SNSCC) is the predominant histological subtype of sinonasal malignancies, comprising about 61% of cases. SNSCC generally adopts a multimodal treatment strategy, primarily surgical intervention, complemented by postoperative radiotherapy and/or chemotherapy (1). In contrast, sinonasal lymphoma (SNL) is more common in China and ranks as the second most frequent primary malignant tumor in the sinonasal area, following squamous cell carcinoma. The principal treatment for SNL involves chemotherapy, supplemented by radiotherapy and immunotherapy, usually excluding surgical options (2). Hence, precise differentiation between these two conditions is essential prior to commencing treatment (3).

The clinical presentations of these diseases are often non-specific, manifesting early symptoms like nasal bleeding and sinusitis. Nasal endoscopy biopsy, while the gold standard for diagnosing both types of tumors, has limited capability in assessing the internal heterogeneity and involvement of adjacent structures (4). Presently, CT and MRI stand as the principal non-invasive imaging techniques for assessing and diagnosing sinonasal disorders. The imaging features and enhancement patterns of SNSCC and SNL on CT and MRI are almost indistinguishable, complicating their differentiation based solely on conventional imaging (5, 6). Thus, developing a non-invasive, comprehensive, and effective early diagnostic method to distinguish between SNSCC and SNL before surgery is crucial for strategic treatment planning.

Machine learning (ML) is extensively utilized in diagnosing head and neck diseases, enhancing clinical decision-making, especially within the anatomically complex sinonasal region (7). Previous research has demonstrated that traditional radiomics models significantly enhance the diagnostic differentiation of SNSCC and SNL (8). Additionally, machine learning can distinguish between various pathological subtypes of SNL (9). However, traditional machine learning approaches often require manual design and feature extraction, which may not thoroughly or accurately assess the complete biological characteristics of the tumor (10).

Deep learning (DL), a subset of machine learning, empowers computers to autonomously learn pattern features and incorporate feature learning into the model development process. This approach mitigates the limitations associated with manual feature design and enhances the accuracy of medical image classification and its broader applicability. Unlike radiomics, convolutional neural networks (CNNs) possess multiple hidden layers that engage with predefined non-linear functions, learning features that surpass traditional radiomics in performance (11, 12). DL based on MRI has been extensively applied in diagnosing and treating SNSCC (10, 13, 14).

Extensive research indicates that ML and DL significantly enhance the segmentation, classification, and prediction of nasopharyngeal carcinoma (15, 16). Feature integration, which combines ML with DL, is an effective strategy that merges manually extracted radiomic features with automatically learned DL features to create a more comprehensive feature set. This method of feature integration maximizes the advantages of traditional features in specific domains while addressing the limitations of deep learning models in handling small datasets, thereby enhancing the diagnostic performance of diseases. So is it possible to combine traditional machine learning algorithms with deep learning techniques in the diagnosis of nasal sinus tumors in order to improve the ability to differentiate squamous cell carcinoma of the nasal sinuses from lymphoma of the nasal sinuses? To date, no research has integrated MRI-based machine learning with three-dimensional (3D) deep learning to differentiate between SNSCC and SNL. This study aims to assess the efficacy of CML models, DL models, and their integrated forms, and to evaluate their effectiveness in the preoperative differentiation of SNSCC from SNL lesions.

The structure of this paper is organized as follows: The first part reviews the diagnostic and treatment strategies for SNSCC and SNL, emphasizing the importance of accurate differentiation and proposing the use of ML and DL technologies to enhance diagnostic performance. The second part describes the research methodology in detail, including data collection, feature extraction, and model construction. The third part presents the research results to validate the effectiveness of the models. The fourth part discusses the findings of the study. Finally, the paper concludes with the main discoveries and suggests directions for future research.

### 1.1 Main contributions

The advantages of feature fusion are evident: This research, through pre-fusion and post-fusion strategies, has validated the efficacy of integrating radiomics and deep learning features, effectively distinguishing between SNSCC and SNL.

Clinical decision support tools have been provided: Based on the study’s findings, a deep learning-radiomics nomogram has been constructed to aid physicians in clinical decision-making.

## 2 Materials and method

### 2.1 General data

**Figure 1:**
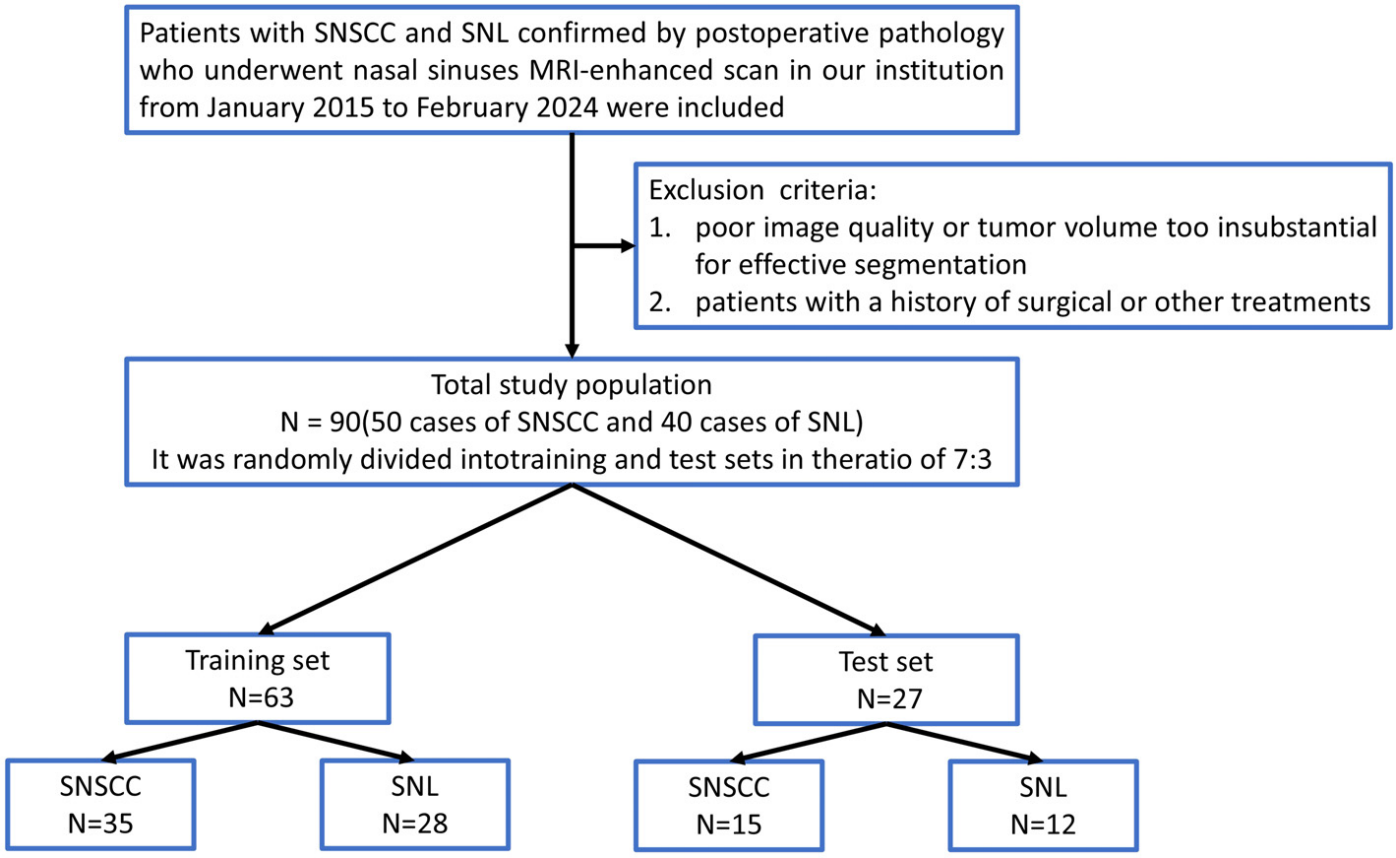
Flowchart of patient recruitment. SNSCC, Sinonasal squamous cell carcinoma; SNL, sinonasal lymphoma.

This retrospective study was approved by our hospital institutional review board. A retrospective analysis was performed on 90 patients diagnosed with sinonasal tumors (mean age, 59.94 ± 14.78 years; 55 males and 35 females; 50 cases of SNSCC and 40 cases of SNL) from January 2015 to September 2024. Clinical data, including age, gender, and smoking history, were collected. Inclusion criteria encompassed: (1) patients confirmed to have SNL or SNSCC through postoperative pathology; (2) patients who had undergone preoperative enhanced MRI scans without prior treatment. Exclusion criteria included: (1) poor image quality or tumor volume too insubstantial for effective segmentation; (2) patients with a history of surgical or other treatments. The recruitment pathway is shown in **Figure 1**.

### 2.2 Instruments and equipment

A Philips 3.0 T superconducting MRI scanner (Achieva TX; the Netherlands), equipped with a 16-channel head and neck coil, was employed. DCE-T1WI scanning parameters were set as follows: 10 dynamic scans per patient, with a TR of 3.3 ms, TE of 1.6 ms, slice thickness of 1 mm, matrix of 244×188, and a field of view (FOV) of 220mm×220mm. The contrast agent utilized was Gd-DTPA (Bayer Healthcare Pharmaceuticals, Germany), administered at a dosage of 0.1 mmol/kg and a flow rate of 2 ml/s.

### 2.3 MRI acquisition and segmentation

Initially, image formats were converted from DICOM to NIFTI, and all images underwent standardization with a pixel spacing adjusted to 1 × 1 × 1 mm³. Regions of interest (ROI) were manually outlined by a radiologist with 10 years of experience using ITK-SNAP 3.8.0 (http://www.itksnap.org) and independently verified by another radiologist with 15 years of experience. Both agreed that the optimal enhancement effect was observed in the lesions of the 7th phase, with the best observation and outlining of the solid components of the lesion, effectively avoiding adjacent nasal sinus tissue. These enhanced lesions on each slice of the 7th phase of the DCE sequence were meticulously delineated, and the data were stored as 3D volumes of interest (VOI).

### 2.4 Model construction and experimental design

This study aimed to develop three types of models based on DCE sequences:

1. Task 1: Extracting radiomics features to construct three classical machine learning (CML) models: logistic regression (LR), k-nearest neighbors (KNN), and light gradient boosting machine (LightGBM);
2. Task 2: Developing three 3D deep learning models (ResNet 10, ResNet 18, and ResNet 34);
3. Task 3: Creating two types of fusion models, specifically feature fusion models and predictive score fusion models.

**Figure 2:**
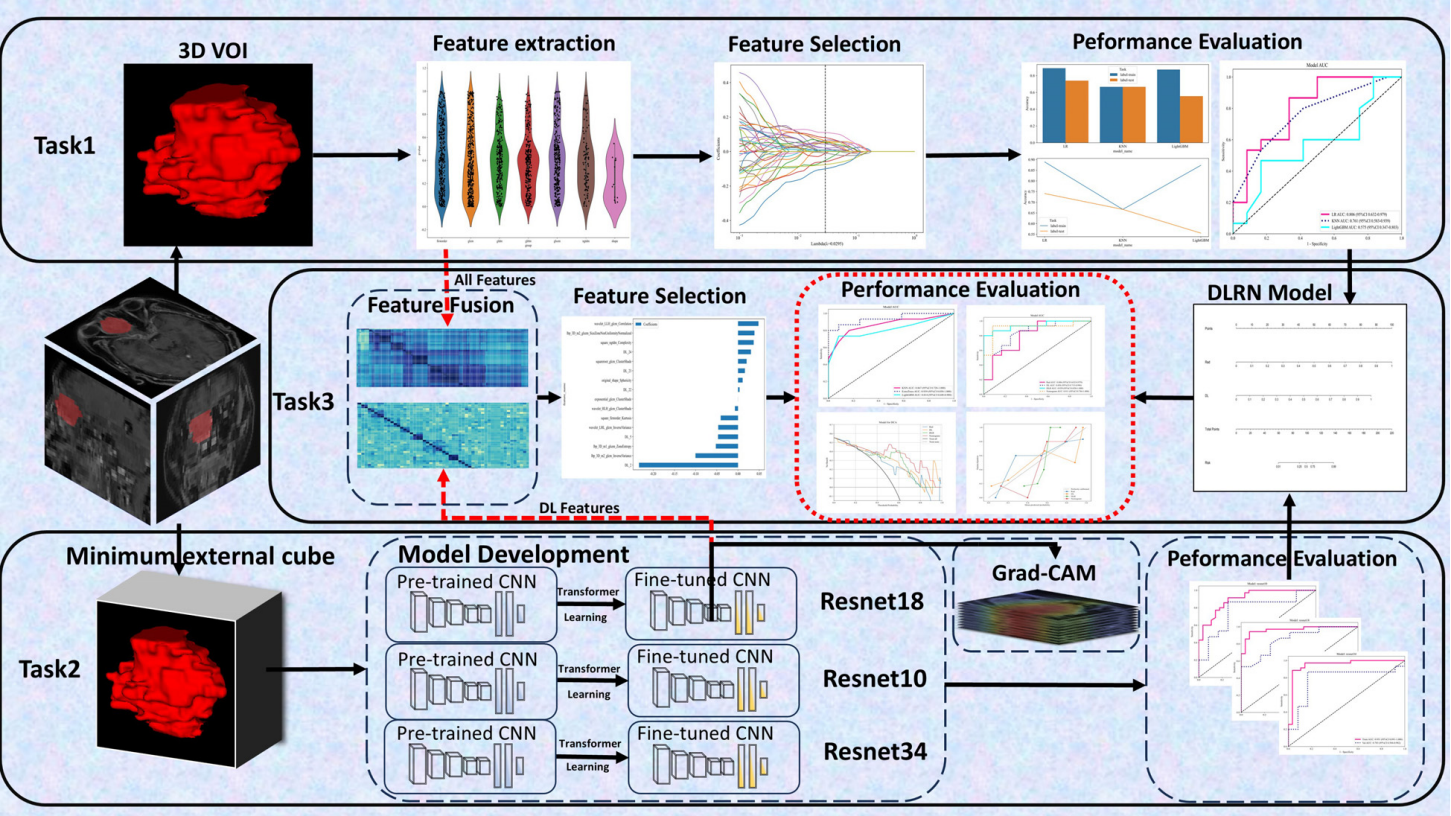
Workflow Diagram. It involves defining 3 tasks using the DCE sequence: (1) Task 1 established 3 CML models; (2) Task 2 developed 3 3D deep learning models; (3) Task 3 created 2 fusion models.

The feature fusion model integrated selected radiomics and deep learning features, utilizing deep learning techniques to formulate a deep learning-based radiomics (DLR) fusion model. This approach combined CML scores and DL scores to develop a predictive score fusion model, termed the deep learning radiomic nomogram (DLRN). The research methodology is depicted in **Figure 2**.

Task 1: Construction and Validation of CML Models

Image preprocessing and feature extraction were conducted using PyRadiomics 3.0.1, resulting in the extraction of 1834 radiomics features. Patients were allocated into training and internal validation sets at a 7:3 ratio. All radiomics features underwent Z-score normalization and t-tests, retaining only those with p-values < 0.05. Pearson correlation coefficients assessed correlations among highly repetitive features; features with coefficients greater than 0.9 were consolidated, retaining only one. The remaining CML features underwent further selection using the Least Absolute Shrinkage and Selection Operator (LASSO) technique.

Classification models were developed for each feature set using CML techniques (LR, KNN, LightGBM). ROC curves were plotted for both the training and validation sets, calculating the area under the curve (AUC), accuracy (ACC), and other metrics to assess each model’s predictive capacity.

Task 2: Construction and Validation of Deep Learning Models

This study was executed in Python 3.10. Initially, all images and delineated ROIs were uploaded into the system, and the minimal bounding box around the tumor was segmented. Surrounding background tissue was removed, isolating the 3D volume of interest (VOI) for model development and testing. 3D-CNNs were utilized to exploit the volumetric data (e.g., MRI images) more thoroughly than 2D and 2.5D methods, enhancing the detection of complex features in supervised learning tasks and bolstering the efficacy of end-to-end automatic disease classification and diagnostic support. This approach deepened the model’s comprehension and analytical capabilities regarding tumors (17). Pre-trained 3D-CNN models (ResNet 10, ResNet 18, and ResNet 34) from the ImageNet dataset were employed for transfer learning. The 3D spatial dimensions of the input VOI were set to 64×64×48. The Adam optimizer, with a learning rate of 0.01 and a batch size of 4, was used. Each model underwent 60 epochs of training on the entire dataset to enhance data interpretation and predictive accuracy, thereby improving the model’s generalization capacity. Adam, a renowned stochastic gradient descent optimization algorithm, integrates adaptive learning rate mechanisms and momentum to optimize neural network parameters, enhancing training efficacy and convergence speed.

Post-training, network parameters were fixed, and the stabilized model served as a feature extractor. DL feature extraction code was derived from the “One-key AI” platform based on PyTorch 1.8.0 (https://github.com/OnekeyAI-Platform/onekey).

Task 3: Development of DLR and DLRN Models

DL features were harvested from the penultimate layer of the finely-tuned network for each patient in both the training and validation cohorts. Information derived from the last convolutional layer was utilized for weighted fusion, employing Gradient-weighted Class Activation Mapping (Grad-CAM) to visualize the model. This process produced class activation maps that underscored critical regions within the images for classification purposes.

Due to the extensive number of features extracted by the DL model, Principal Component Analysis (PCA) was implemented to manage the high-dimensional data. PCA, a statistical method, simplifies data by discerning patterns and interrelationships among variables within high-dimensional datasets, thus minimizing redundant information and enhancing the efficiency of subsequent data analysis and modeling (18). The PCA-reduced features were amalgamated with radiomics features to advance DLR modeling. The feature selection procedure and model development approach for the DLR model mirrored those used for the CML models, leading to the establishment of three models: KNN, ExtraTrees, and RandomForest. In this model, we fuse radiomics features with deep learning extracted features in the same feature space. Such fusion can fully utilize the advantages of both and mitigate the instability linked to constrained sample sizes.

Moreover, a predictive score fusion model, known as DLRN, was devised by amalgamating the respective CML and DL scores. The DLRN was engineered by weighting these scores based on their respective coefficients to compute the DLRN for each patient across the training and validation datasets. In this model, we train CML and DL models separately and then combine their outputs. By combining the predictions of different models, this strategy is able to reduce the possible bias of a single model and improve the robustness and accuracy of the overall classification.

The optimal CML model, premier DL model, leading DLR model, and the DLRN model were all evaluated and their performances compared using AUC among other metrics. Decision Curve Analysis (DCA),calibration curves and Delong test were employed to assess the clinical utility of these models.

### 2.5 Statistical analysis

The clinical characteristics of the training and validation cohorts were analyzed using Python 3.7.0 and the statsmgdels version 0.13. Continuous variables were assessed using independent sample t-tests or Mann-Whitney U tests, and categorical variables were examined via chi-square tests. A p-value of less than 0.05 was deemed to indicate statistical significance.

## 3 Results

### 3.1 Clinical data

A total of 73 patients were included in this study. The patients were randomly divided into training (n = 51, SNSCC = 26 and SNL = 25) and validation (n = 22, SNSCC = 16 and SNL = 6) sets in a 7:3 ratio. There were no statistically significant differences in age, gender, or smoking history between the two groups (P > 0.05 for all, as shown in **Table 1**).

**Table 1:** Clinical information of SNSCC and SNL patients in this study.

Clinical Characteristics	Training Set – ALL(n=63)	Training Set – SNL(n=28)	Training Set – SNSCC(n=35)	P Value	Validation Set – ALL(n=27)	Validation Set – SNL(n=12)	Validation Set – SNSCC(n=15)	P Value
**Age**	59.94 ± 14.78	57.71 ± 15.72	61.71 ± 13.95	0.289	58.37 ± 12.70	58.50 ± 10.34	58.27 ± 14.68	0.963
**Gender**				1.0				0.964
**Male**	38(60.32)	17(60.71)	21(60.00)		17(62.96)	7(58.33)	10(66.67)	
**Female**	25(39.68)	11(39.29)	14(40.00)		10(37.04)	5(41.67)	5(33.33)	
**Smoking History**				1.0				
**Non-smoker**	29(46.03)	13(46.43)	16(45.71)		11(40.74)	5(41.67)	6(40.00)	1.0
**Smoker**	34(53.97)	15(53.57)	19(54.29)		16(59.26)	7(58.33)	9(60.00)	

### 3.2 Task 1: Construction and validation of CML models

**Figure 3:**
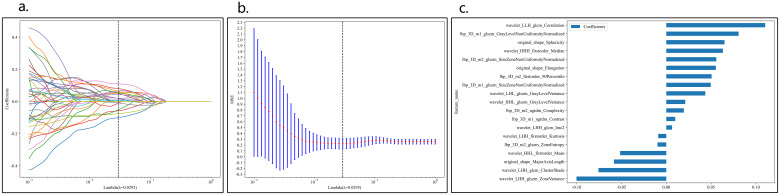
Coefficients of 10-fold cross-validation in CML model **(A)**. MSE of 10-fold cross-validation in CML model **(B)**, λ=0.0596. Selected features weight coefficients **(C)**.

A total of 1834 features were extracted across 7 categories, including 14 shape features, 360 first-order features, and 1460 texture features. After LASSO regression screening, 19 non-zero coefficient features were selected for further analysis. The coefficients and mean squared error (MSE) from 10-fold cross-validation are shown in **Figure 3**.

**Figure 4:**
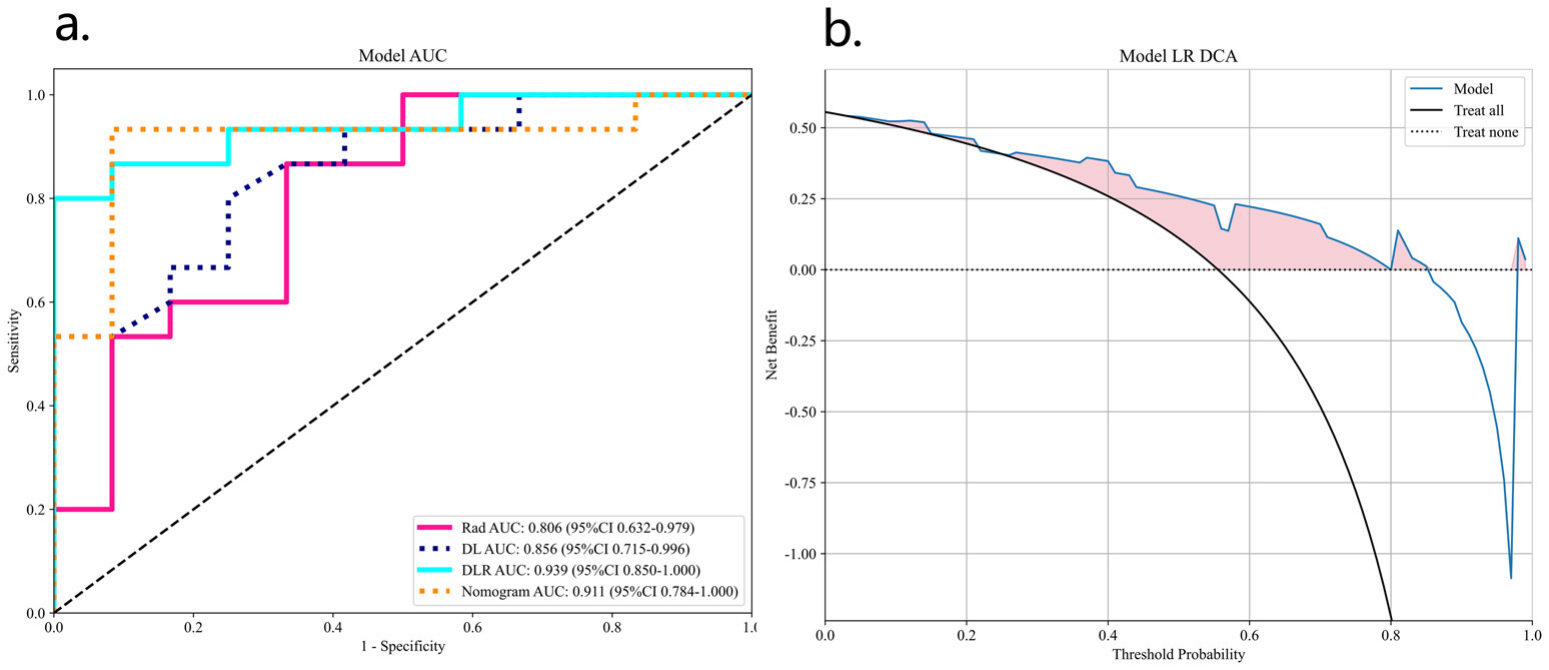
ROC analyses for different CML model validation groups **(A)** and DCA curves for the best model LR **(B)**.

**Table 2** presents the performance of the CML models, with LRoutperforming the KNN and LightGBM classifiers. The AUC values for theLR model in the training and validation sets were 0.967 and 0.806, respectively. Additionally, the DCA curve for the best model, KNN, in the validation set is shown in **Figure 4**.

**Table 2:** Performance of each model on the training and validation sets.

Model Type	Model Name	AUC	95% CI	Accuracy	Sensitivity	Specificity	PPV	NPV	Group
**CML Models**	LR	0.967	0.9330- 1.0000	0.889	0.800	1.000	1.000	0.800	**Training Set**
	**LR**	**0.806**	0.6319– 0.9792	0.741	0.800	0.667	0.750	0.727	**Validation Set**
	LightGBM	0.925	0.8590- 0.9910	0.873	0.829	0.929	0.935	0.812	**Training Set**
	LightGBM	0.575	0.3471- 0.8029	0.556	0.333	0.833	0.714	0.500	**Validation Set**
	KNN	0.922	0.8624- 0.9815	0.667	0.400	1.000	1.000	0.571	**Training Set**
	KNN	0.761	0.5833- 0.9389	0.667	0.533	0.833	0.800	0.588	**Validation Set**
**DL Models**	ResNet10	0.906	0.8347-0.9756	0.825	0.886	0.750	0.816	0.840	**Training Set**
	ResNet10	0.772	0.5808-0.9637	0.778	0.800	0.750	0.800	0.750	**Validation Set**
	ResNet18	0.966	0.9267-1.0000	0.921	0.914	0.929	0.941	0.897	**Training Set**
	**ResNet18**	**0.856**	0.7153-0.9958	0.741	0.733	0.750	0.786	0.692	**Validation Set**
	ResNet34	0.951	0.8911-1.0000	0.905	0.857	0.964	0.968	0.844	**Training Set**
	ResNet34	0.783	0.5843-0.9824	0.815	0.800	0.833	0.857	0.769	**Validation Set**
**DLR Models**	KNN	0.997	0.9920- 1.0000	0.921	0.857	1.000	1.000	0.848	**Training Set**
	KNN	0.867	0.7281- 1.0000	0.778	0.667	0.917	0.909	0.687	**Validation Set**
	ExtraTrees	0.995	0.9853- 1.0000	0.952	0.914	1.000	1.000	0.903	**Training Set**
	**ExtraTrees**	**0.939**	0.8501- 1.0000	0.852	0.733	1.000	1.000	0.750	**Validation Set**
	LightGBM	0.931	0.8714- 0.9909	0.857	0.800	0.929	0.933	0.788	**Training Set**
	LightGBM	0.814	0.6478- 0.9800	0.667	0.400	1.000	1.000	0.571	**Validation Set**
**DLRN Model**		0.995	0.9841 - 1.0000	0.968	0.971	0.964	0.971	0.964	**Training Set**
		**0.911**	**0.7845 - 1.0000**	**0.889**	**0.867**	**0.917**	**0.929**	**0.846**	**Validation Set**

The AUC value of the model with the best efficacy in each task was bolded, and all the performances of the best performing DLRN model were bolded.

### 3.3 Task 2: Construction and validation of deep learning models

**Figure 5:**
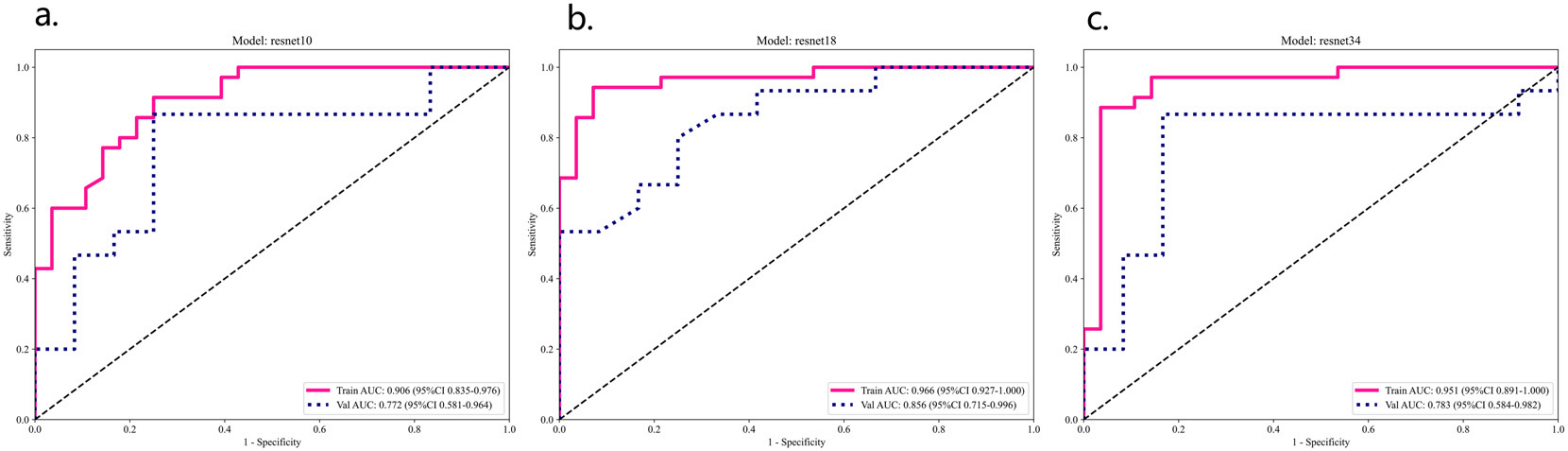
ResNet 10 **(A)**, ResNet 18 **(B)**, ResNet 34 **(C)** model training set and validation ROC analysis.

In the validation set, the ResNet 18 model demonstrated superior performance compared to the other two deep learning models (see **Table 2**; **Figure 5**). The ResNet 18 model achieved an AUC of 0.856, an accuracy of 0.741, a sensitivity of 0.733, and a specificity of 0.750 in the validation set.

### 3.4 Task 3: Construction of DLR and DLRN models

Due to the better predictive performance of the ResNet 18 model, we extracted deep learning features from the fixed ResNet 18 model. A total of 512 DL features were extracted from each 3D sample and reduced to 32 DL features using PCA. The DL score consists of these 32 features.

**Figure 6:**
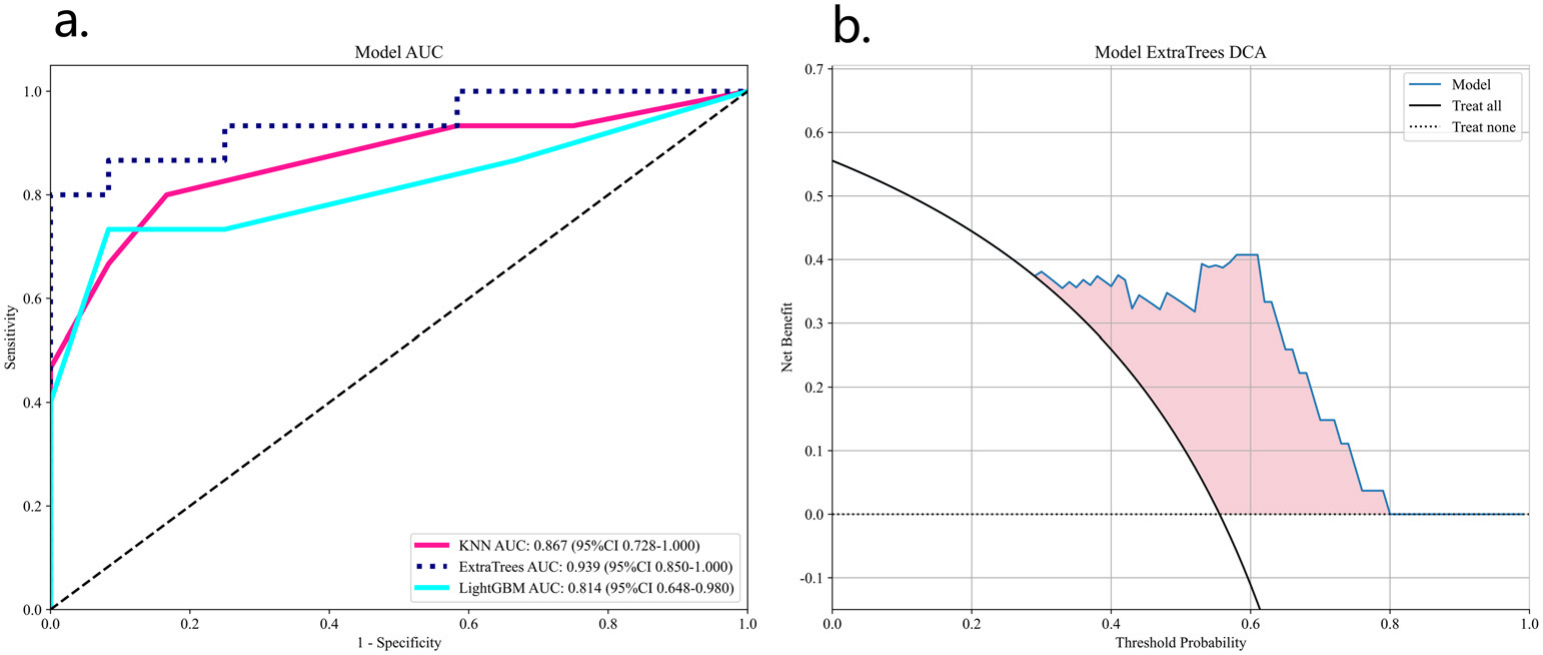
ROC analysis of three DLN models validation group **(A)** and DCA curves for the best model ExtraTrees **(B)**.

These 32 DL features were combined with the 1834 radiomics features from Task 1, resulting in a total of 1866 features. After LASSO logistic regression screening, only 16features were retained. Finally, due to the superior performance of the ExtraTrees classifier model (**Table 2**), we used the ExtraTrees classifier to construct the DLR model (**Figure 6**).

**Figure 7:**
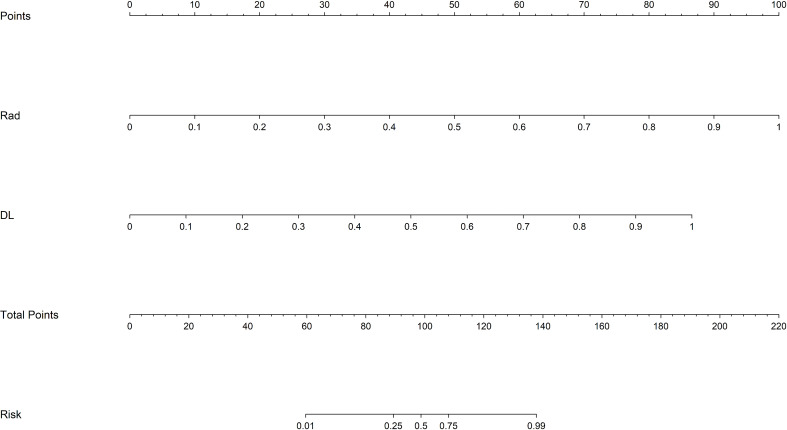
DLRN model. The predicted predictor values (ResNet 18 signature and CML-LR signature) can be transformed into risk points. The sum of the risk points of the predictors on the total score axis can then be mapped to the risk axis to derive the probability of SNSCC.

We also constructed a feature-selected predictive score fusion model (DLRN). The DLRN framework is based on the combination of CML scores and DL scores. The individual variable values (KNN score and ResNet 18 score) were determined based on the top Points scale, and the points for each variable were summed (**Figure 7**). Finally, the probability of diagnosing SNSCC was obtained using the Total Points scale at the bottom.

**Table 2** lists all the models used for differentiating SNSCC and SNL. The ExtraTrees classifier in the DLR model has the highest AUC values in the training and validation sets of 0.995 and 0.939, respectively. The DLRN model, although its validation set AUC value (AUC=0.911) is slightly lower than that of the best DLR model, its validation set accuracy and sensitivity (accuracy of 0.889 and sensitivity of 0.867) are higher than all other models, which shows the highest performance.

**Figure 8:**
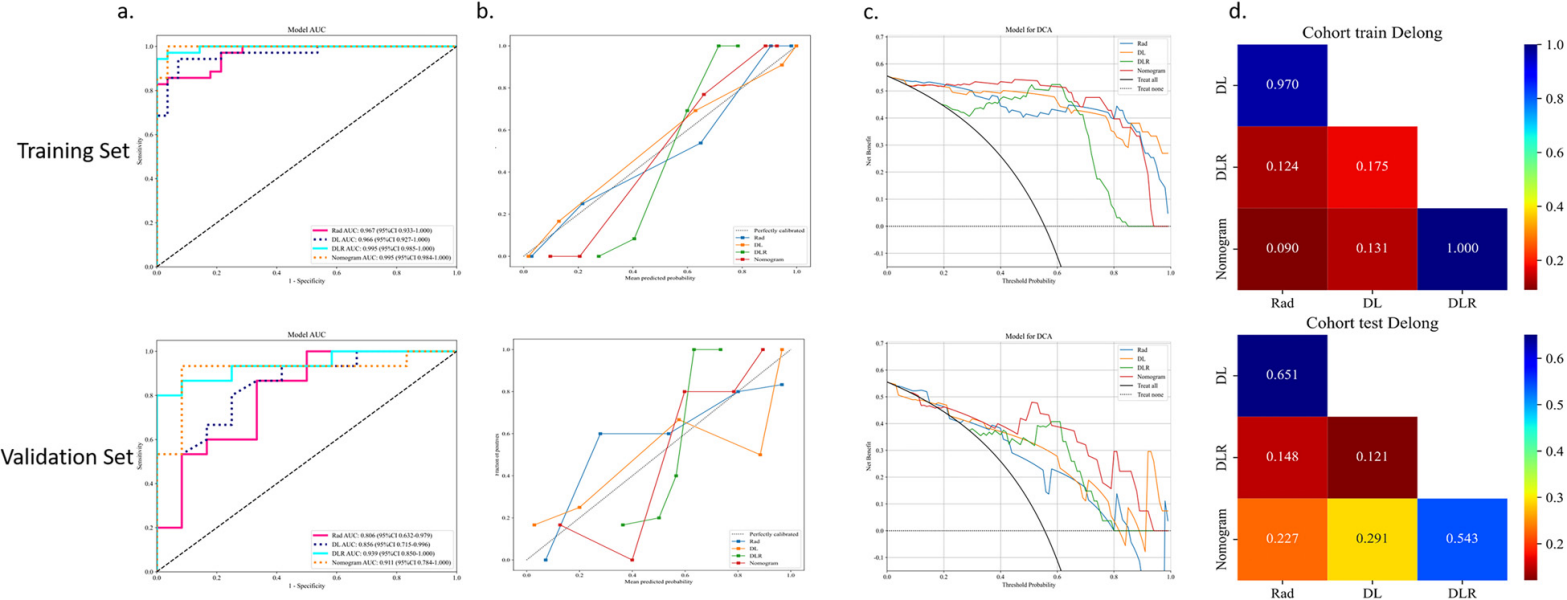
The summary of the best CML model, best DL model, best DLR model, and DLRN model in terms of ROC **(A)**, calibration curves **(B)**, DCA curves **(C)**, and Delong test **(D)** on training and validation cohorts is as follows.

**Figure 8** shows the AUC, calibration curves, DCA and Delong test for the best CML model (KNN), best DL model (ResNet18), best DLR model, and DLRN model in the training and validation cohorts. During training, the preoperative application of the DLRN model demonstrated higher clinical benefits in distinguishing SNSCC from SNL compared to the other three models.

## 4 Discussion

Sinonasal squamous cell carcinoma (SNSCC) and sinonasal lymphoma (SNL) are the two most prevalent malignant tumors in the sinonasal region, yet their etiologies remain elusive. They may be linked to exposure to industrial substances like formaldehyde, wood dust, welding fumes, as well as smoking and tobacco use (1, 19). Consequently, this study incorporated an analysis of the smoking history of patients with these conditions. Given the limited availability of clinical factors and the absence of statistical significance, clinical factors were not included in this study, aligning with prior research (8, 13).

The choice to base this study on a single DCE-T1WI sequence was driven by several factors. Previous research on head and neck tumors has demonstrated that DCE-MRI holds promising potential for diagnosing and treating squamous cell carcinoma and lymphoma, especially in differentiating between the two (20, 21). Research has shown that, compared to other standard MRI sequences, the texture parameters of DCE-T1WI significantly expose the internal heterogeneity and structure of tumors (8, 22, 23). Su et al. (24) analyzed the texture parameters of T2WI, ADC, and DCE-T1WI images in patients with sinonasal non-Hodgkin lymphoma (NHL) and squamous cell carcinoma (SCC), finding that the texture parameters based on the T2WI sequence lacked statistical significance, while the mean value of DCE-T1WI in SCC was substantially higher than in NHL (p=0.015). This indicates that the DCE sequence offers higher specificity in texture features compared to other sequences, and enhanced scanning aids in delineating the extent of lesion infiltration.

Although previous studies have acknowledged the diagnostic value of CT or MRI in identifying lymphoma and squamous cell carcinoma, traditional imaging features often do not distinctly differentiate between the two (5, 6). It has been noted that diffusion-weighted MRI (DWI) can enhance the accuracy of distinguishing these tumors in the head and neck area. However, due to the similar histological characteristics shared by squamous cell carcinoma and lymphoma, their ADC values may closely align in certain cases, resulting in reduced specificity in differentiating ADC values (25). Consequently, features that are visually identifiable may not accurately reflect the pathological details of SCC or lymphoma. Radiomics, on the other hand, transforms images into high-throughput quantitative features that thoroughly characterize the potential heterogeneity of tumors, thereby facilitating clinical decision-making. Numerous studies have demonstrated that integrating machine learning with omics can significantly improve the accuracy of omics data, offering great potential in disease classification and diagnosis (26). Wang et al. (8)developed an MRI-based radiomics model using a support vector machine (SVM) classifier to distinguish SNL from SNSCC, achieving AUC values of 0.94 and 0.85 in the training and validation sets, respectively, proving that radiomics can more precisely differentiate SNL from SNSCC. In our analysis, among the CML models created based on the DCE-T1WI sequence, the Logistic Regression (LR) algorithm emerged as the most effective classifier, registering the highest AUC in both the training and validation sets (AUC=0.806).

Deep learning algorithms have significantly impacted image recognition, disease diagnosis, and prognosis prediction (27, 28). CNNs have enhanced medical imaging, but increasing neural network layers can cause issues such as gradient vanishing or network degradation, which may reduce performance. We selected ResNet for this study because, unlike traditional non-residual deep learning networks, it effectively addresses the issues of gradient vanishing and performance degradation through the introduction of residual connections. This allows ResNet to maintain better stability, efficiency, and performance in training deep networks (29). In this research, the ResNet 18 classifier demonstrated the highest performance (AUC=0.856), while the ResNet 10 classifier showed the lowest. The performance variance among the DL models can be attributed to their differing network depths and complexities (30). Mohammeda et al. (31) proposed a new method for the automatic segmentation and recognition of nasopharyngeal carcinoma microscopic images based on artificial neural networks. The results indicate that neural network classifiers outperform SVM classifiers in handling high-dimensional features. Our findings also show that DL models are more effective than CML models, likely because deep learning enables end-to-end classification and prediction, automatically extracting complex features from the raw pixels of input images without relying on manually designed feature extraction methods (32).

The advantage of CML lies in its ability to reduce data complexity through dimensionality reduction techniques; deep learning excels at capturing deeper features. Integrating traditional radiomics with deep learning for feature integration not only reduces biases and overfitting from single feature sets, thus enhancing model robustness, but also introduces more diverse information, improving the model’s adaptability across different scenarios. Studies have shown that combining these two model types yields better results in differential diagnosis of head and neck diseases than using a single model (33, 34). **Table 2** lists all models differentiating SNSCC from SNL, with the ExtraTrees classifier in the DLR model achieving the highest AUC values in both training and validation sets. In the DLR model, the ExtraTrees classifier outperformed the other two classifiers, likely due to its ability to automatically perform feature selection with tree models, effectively handling high-dimensional feature data and minimizing the impact of feature quantity on model performance. Although the DLRN model has a slightly lower AUC in the validation set compared to the best DLR model, its accuracy and sensitivity are higher than all other models. The DLRN model not only exhibited the highest performance but also provided a visual tool that transforms complex statistical data into simple graphical representations, offering clinicians a method for quantitative assessment of diagnoses. Overall, the integration of DL and CML technologies holds the potential to non-invasively differentiate SNSCC from SNL.

This study has several limitations. (1) The relatively small sample size from a single-center study may affect the model’s generalizability. (2) To ensure more robust and reliable analysis, a larger dataset is necessary. Although feature integration reduces the risk of overfitting associated with single feature sets, overfitting may still occur with increased model complexity. (3) This study focused solely on modeling DCE-MRI sequences without comparing other sequences. (4) Manual segmentation was used instead of automatic segmentation, which is time-consuming and susceptible to subjective biases, potentially leading to poor segmentation consistency. (5) Due to the limited sample size, we distinguished only between all lymphomas and squamous cell carcinomas without further subgroup analysis of different lymphoma subtypes.

## 5 Conclusion

This paper discusses the application value of integrating radiomics and deep learning features in differentiating SNSCC from SNL. The findings indicate that the feature integration models based on DCE outperform those using features independently. Among these, the post-fusion model (DLRN), which integrates CML and DL scores into a nomogram, demonstrated superior performance, effectively distinguishing between SNSCC and SNL with significant potential for clinical decision support.

The radiomics feature integration approach used in this study captures complementary information from different data types, reducing biases associated with single features, thereby maintaining good performance and stability under various conditions, thus enhancing the accuracy of disease diagnosis and providing a scientific basis for personalized treatment plans.

Future studies should explore automatic segmentation algorithms for nasal and paranasal sinus tumors to overcome the limitations of manual extraction. To overcome the limitations of a single theoretical model, it is necessary to expand the sample size and integrate other MRI sequences or omics data. Beyond differential diagnosis between the two nasal conditions, the applicability of this method in other head and neck diseases should be considered. The complex anatomical structure of the head and neck and the interplay of various diseases highlight the broad application potential of the feature integration approach.

## Data Availability

The original contributions presented in the study are included in the article/supplementary material. Further inquiries can be directed to the corresponding author/s.
